# Pediatric Parapharyngeal Space Mass: Successful Outcome Following Transoral Simple Cyst Resection

**DOI:** 10.1155/crot/9934709

**Published:** 2025-10-29

**Authors:** Christian M. Kabongo, Alexander P. Marston

**Affiliations:** ^1^Tufts University School of Medicine, Boston, Massachusetts, USA; ^2^Department of Otolaryngology-Head and Neck Surgery, University of California Davis Health, Sacramento, California, USA

## Abstract

**Background:**

Parapharyngeal space masses are rare lesions identified in pediatric patients. The present case describes the clinical history and surgical treatment of a parapharyngeal space in a 32-month-old female.

**Methods:**

The electronic medical record was used for this retrospective case report.

**Results:**

A 32-month-old female had a nonsignificant clinical presentation and a negative flexible laryngoscopy. Upon imaging, a mass was seen in the left parapharyngeal space. Resection of the mass was conducted. The final pathology confirmed a benign simple cyst. The patient did not have any postoperative complications.

**Conclusions:**

This case illustrates that surgical resection can be an adequate treatment method for a parapharyngeal space mass. It also adds to the literature on types of parapharyngeal space masses that may present in pediatric patients.

## 1. Introduction

Parapharyngeal space masses are rare, typically benign, and range from glioneuronal heterotopias to sarcomas. They are especially uncommon in pediatric patients, with recent studies, such as Starek et al., documenting only 20 cases, while an independent literature review for this report identified 31 [1]. Pediatric presentations vary, including odynophagia or simply incidental findings on imaging [1]. Due to their location, these masses can significantly impact swallowing and respiration, affecting quality of life as the child develops. Given the limited number of reported cases, no standardized approach to characterization or treatment has been established [1]. Given their potential to impair quality of life, identifying the most effective treatment strategies is crucial. This report discusses a case of a benign simple cyst in the left parapharyngeal space of a 32-month-old female, successfully treated with gross resection.

## 2. Case Presentation

**Figure 1:**
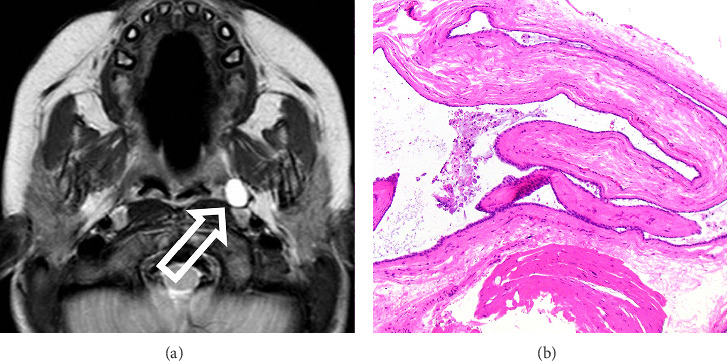
T2 noncontrast axial MR image displaying a hyperintense lesion in the left parapharyngeal space (a). Histologic image at 100x with hematoxylin and eosin stain demonstrating short cuboidal epithelium with abundant extravasated mucin, consistent with a benign simple cyst (b).

A 32-month-old female was referred to the Pediatric Otolaryngology clinic due to an incidentally identified parapharyngeal space mass on magnetic resonance (MR) imaging. Cervical imaging was previously obtained due to an exam finding of kyphosis, which demonstrated congenital vertebral fusion of L3-L4. The MR also revealed a T2 hyperintense lesion measuring 9 mm in diameter within the left parapharyngeal space (Figure 1). The patient had no symptoms of dysphagia, snoring, or respiratory difficulty. There was a history of congenital vertebral fusion in the patient's maternal grandmother, but the remainder of the family history was unremarkable.

Oropharyngeal examination and flexible fiberoptic nasopharyngoscopy were within normal limits. The cervical exam was negative for masses or lymphadenopathy.

Options of serial MR imaging for surveillance of the parapharyngeal space lesion versus surgical excision were offered to the family. Surgery was selected, and the team proceeded with a unilateral left-sided extracapsular tonsillectomy and transoral parapharyngeal space exploration for complete mass excision.

## 3. Discussion

The parapharyngeal space is defined by the skull base superiorly, hyoid bone inferiorly, parotid and masticator spaces laterally, and the deep cervical fascia investing the pharyngeal constrictor muscle medially. The space can be further categorized into prestyloid and poststyloid compartments. The prestyloid parapharyngeal space includes fat, minor salivary glands, internal maxillary artery, and mandibular branches of the trigeminal nerve. The poststyloid compartment includes the internal jugular vein, carotid artery, sympathetic chain, and cranial nerves IX through XII. Parapharyngeal space masses are primarily identified in adults and are uncommon in pediatric patients [2]. When identified in pediatric patients, the diagnosis is typically incidental in nature. However, if the mass is large in size, pediatric patients may present with symptoms such as snoring, nasal congestion, and dysphagia [3–6]. Parapharyngeal space masses in adults are reported to be of malignant pathology in approximately 20% of cases, whereas in pediatric patients, malignancy is reported in up to two-thirds of presentations [1, 2]. In the published English-based literature between 1999 and 2025, a total of 31 pediatric parapharyngeal space masses were reported. These were derived from the following pathologies: glioneuronal heterotopia (45.2%), branchial cleft cysts (9.7%), ganglioneuromas (16.1%), fibromas (3.2%), sarcomas (9.7%), adenomas (3.2%), ectomesenchymomas (3.2%), hemangiomas (3.2%), lipoblastomas (3.2%), and schwannomas (3.2%) [1–10]. The mean age at the time of diagnosis for parapharyngeal space masses in pediatric patients was reported to be 3.7 years [1–10].

MR imaging is the ideal imaging modality used in evaluating and characterizing parapharyngeal space masses [6]. The MR findings in the presented case demonstrated a T2 hyperintense, T1 hypointense, and nonenhancing prestyloid parapharyngeal space lesion located just deep to the superior pharyngeal constrictor muscle adjacent to the palatine tonsil (Figure 1). The T2 hyperintense features of the mass in this case correlated with a benign simple cyst, lined by short cuboidal epithelium with abundant extravasated mucin, as confirmed by pathologic analysis (Figure 1). Based on a review of the literature, the presented case is the only description of a simple benign cyst of the parapharyngeal space in a pediatric patient. Since the prestyloid compartment includes minor salivary glands, the origin of this mass may be salivary in nature; however, no definitive salivary architecture was identified in the histopathologic analysis.

**Figure 2:**
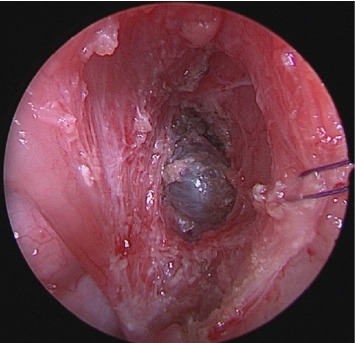
Following extracapsular tonsillectomy and dissection through the superior pharyngeal constrictor muscle, the benign simple cyst of the parapharyngeal space is visualized.

Parapharyngeal space masses present a unique set of treatment challenges that necessitate careful consideration of the size, specific location, and relationship of the mass to surrounding structures. Depending on a patient's presentation, exam and imaging characteristics, observation with interval imaging, biopsy, and complete mass excision can all be considered as treatment options. Since up to two-thirds of pediatric parapharyngeal masses can represent a malignant diagnosis, it is important to follow these lesions and have a low threshold for biopsy and/or removal [3]. When surgery is selected, either transoral or transcervical approaches can be utilized to access the parapharyngeal space [2]. In the case of the presented patient, the small size and location in the prestyloid parapharyngeal space, just deep to the pharyngeal constrictor muscle, allowed for complete removal via a transoral approach. Following transoral extracapsular tonsillectomy, the pharyngeal constrictor muscle was carefully transgressed with subsequent access to the prestyloid parapharyngeal space and localization of the mass (Figure 2). The mass was completely excised via this approach with an uncomplicated recovery. Postoperatively, there was no evidence of recurrence at 6 months following the surgical excision.

## 4. Conclusion

This case uniquely demonstrates an incidentally discovered prestyloid parapharyngeal space mass in a pediatric patient that was completely excised transorally. Although unique tumor pathologies are known to occur in the parapharyngeal space, this report reveals a benign simple cyst and represents a diagnosis that should be considered when encountering T2 hyperintense parapharyngeal space lesions on MR imaging.

## Data Availability

This case report focuses on the work-up and surgical case management within the division of Pediatric Otolaryngology of the Department of Otolaryngology—Head and Neck Surgery at Tufts Medical Center. Radiologic imaging, pathological specimens, and patient clinical history were accessed via the secure electronic medical record system.
